# The PD-1/PD-L1 Gateway: Peripheral Immune Regulation in the Pathogenesis of Endometriosis

**DOI:** 10.3390/ijms25126775

**Published:** 2024-06-20

**Authors:** Małgorzata Sobstyl, Paulina Mertowska, Sebastian Mertowski, Monika Zaborek-Łyczba, Dominik Dudziński, Grzegorz Polak, Ewelina Grywalska

**Affiliations:** 1 Department of Gynecology and Gynecological Endocrinology, Medical University of Lublin, 20-049 Lublin, Poland; malgorzata.sobstyl@umlub.pl; 2 Department of Experimental Immunology, Medical University of Lublin, 20-093 Lublin, Poland; sebastian.mertowski@umlub.pl (S.M.); monika.zaborekk@gmail.com (M.Z.-Ł.); d.dudzinski1996@gmail.com (D.D.); ewelina.grywalska@umlub.pl (E.G.); 3 Independent Laboratory of Minimally Invasive Gynecology and Gynecological Endocrinology, Medical University of Lublin, 20-081 Lublin, Poland; grzegorz.polak@umlub.pl

**Keywords:** PD-1, PD-L1, endometriosis, immune system, lymphocyte T, lymphocyte B

## Abstract

Endometriosis is a chronic inflammatory disease characterized by the presence of endometrial-like tissue outside the uterine cavity, causing pain and infertility. Despite the rather unclear etiopathogenesis, recent studies suggest the involvement of the immune system in the development and progression of endometriosis. The role of the PD-1/PD-L1 axis in the modulation of the immune response in this disease seems to be particularly interesting. This preliminary study aimed to investigate the expression of PD-1 and PD-L1 on T and B lymphocytes in peripheral blood in patients with endometriosis to assess their potential impact on disease progression. Our study involved peripheral blood samples from 80 patients diagnosed with endometriosis and 20 healthy women as a control group were analyzed. Flow cytometry was used to assess the expression of PD-1 and PD-L1 on T and B lymphocytes, and enzyme-linked immunosorbent assays were used to assess their soluble forms in serum and peritoneal fluid.in our research we observe significantly higher expression of PD-1 and PD-L1 on T and B lymphocytes was found in patients with endometriosis compared to the control group. Higher expression of both tested molecules correlated with the stage of endometriosis. The results of our preliminary studies indicate a potential role of the PD-1/PD-L1 axis in the modulation of the immune response in endometriosis. Modified expression of these proteins may contribute to immune evasion by ectopic tissues, supporting their survival and proliferation. These findings suggest that targeting PD-1/PD-L1 could be explored as a therapeutic option for the treatment of endometriosis, though further research with larger sample sizes is necessary to confirm these results and clarify the role of PD-1/PD-L1 in the pathogenesis of the disease.

## 1. Introduction

Endometriosis is one of the most frequently diagnosed gynecological diseases in recent years, characterized by the presence of endometrial-like tissue outside the uterine cavity. This chronic and often painful disease affects the lives of millions of women around the world, but its exact etiology and pathophysiological mechanisms are still not fully understood. Existing evidence suggests that abnormalities in the immune system play a key role in the initiation and progression of endometriosis, particularly through T- and B-cell-mediated immune dysfunction [1,2,3,4,5].

T cells are activated in response to antigens presented by antigen-presenting cells such as dendritic cells. In endometriosis, T cells can be activated by antigens derived from ectopic endometrial tissue. However, as a result of the expression of immunosuppressive molecules such as PD-L1 by ectopic cells, the T cell response can be suppressed, allowing immune destruction by ectopic cells to be avoided [6,7,8,9].

B lymphocytes are responsible for the production of antibodies that are normally used to identify and neutralize pathogens. In endometriosis, antibodies may be produced against cells of ectopic endometrial tissue, which may indicate an autoimmune aspect of the disease. However, the effectiveness of this response is often limited by the local immunosuppressive environment. B lymphocytes, in addition to producing antibodies, can also influence the inflammatory response by producing various cytokines. In this way, they can modulate the activity of other immune cells, including T lymphocytes and macrophages, which may influence the course of endometriosis [10,11].

The immune system’s impact on endometriosis is multidimensional and includes both defensive aspects and aspects that promote the survival and spread of ectopic endometrial cells. A significant element modulating the immune response is PD-1 (programmed cell death protein 1) and its ligand PD-L1 (programmed death-ligand 1). These proteins are important in regulating the immunosuppressive response, especially in the context of maintaining immune tolerance to self-antigens and regulating the anti-inflammatory response. Existing knowledge indicates that in endometriosis, the expression of PD-1 and PD-L1 may be increased. This upregulation can inhibit the cytotoxic activity of T and B lymphocytes, reducing their effectiveness in eliminating ectopic endometrial tissue. However, the extent and precise mechanisms by which PD-1 and PD-L1 contribute to immune evasion in endometriosis remain unclear, forming the basis for further investigation in this study [12,13,14,15].

Therefore, this study aimed to determine the role of the PD-1/PD-L1 axis on T and B lymphocytes in peripheral blood and peritoneal fluid of patients with endometriosis. This analysis aims to better understand the mechanisms of immunosuppression observed in this disease as well as to evaluate potential new therapeutic targets that could contribute to the development of modern therapies.

## 2. Results

### 2.1. Characteristics of the Study Group, i.e., How Can Endometriosis Affect the Basic Parameters of Morphology, Biochemistry, and Immunophenotype of Peripheral Blood?

Our research group conducted intensive research on the development and progression of endometriosis in women in Poland. In this publication, we present research on 80 newly diagnosed and untreated patients with endometriosis, divided into 20 people for each of the stages I–IV. All patients were diagnosed and recruited following the guidelines of the American Society for Reproductive Medicine (ASRM) [16]. The control group consisted of healthy volunteers without signs of endometriosis or other reproductive system disorders. For each patient included in the study (both from the study and control groups), a complete set of tests was performed to analyze selected parameters of peripheral blood morphology and biochemistry, as well as immunophenotypic analyses, including determining the percentage of T and B lymphocytes and assessing the degree of their activation. The obtained results were first analyzed for all patients with endometriosis in the control group (Table 1 and Table 2) and then the individual stages of endometriosis were taken into account (Table 3 and Table 4).

The results presented in Table 1 show that patients with endometriosis included in this study are characterized by an increased number of neutrophils, monocytes, and lymphocytes compared to patients from the control group. Moreover, the concentration of Ca-125 is also significantly higher. The assessment of TSH, FT3, FT4, estradiol, FSH, and LH levels in serum was not statistically significant between patients. Significant changes were observed in immunophenotypic analyses, which showed a significant increase in the activation of the percentage of all tested lymphocytes in patients with endometriosis (Table 2).

More detailed analyses of the parameters discussed above, which were carried out for individual stages of endometriosis, also showed several significant changes (Table 3 and Table 4).

The greatest differences were found in the number of monocytes and lymphocytes in the patient’s peripheral blood, which increased with the change in the stage of the disease. Ca-125 concentration was statistically significant in all stages of endometriosis, except for differences between stages III and IV. HE4 concentration showed a significant difference between stages I and III and I and IV. Analysis detailing individual stages of endometriosis also showed significant differences in the levels of tested hormones. The levels of estradiol, FSH, and LH increased with the severity of the disease and were lower than in healthy volunteers. Significant differences between individual stages were demonstrated for estradiol between I and III; I and IV; II and III; and II and IV; for FSH between I and III; I and IV; and II and IV; and for LH between I and IV; II and III; and II and IV (Table 3).

The analysis of selected immunological parameters in patients with endometriosis, taking into account their stage, also showed significant changes, not only in the results observed in healthy patients but directly between stages. The percentage of CD19+ B cells was significantly different between patients in stages I and IV and III and IV, while the percentage of CD8+ T cells was significantly different between patients in stages I and II; I and III; and I and IV. Particularly noteworthy is the contribution of CD4+CD25+ T cell activation, the observed values of which increased with the development of the endometriosis stage, while the CD4+CD69+ values decreased (the same tendency was observed for CD8+CD69+) (Table 4).

### 2.2. Involvement of the PD-1/PD-L1 Pathway in the Development of Endometriosis

In the next step, we decided to analyze the involvement of PD-1 and PD-L1 molecules in the immunopathogenesis of endometriosis. As in the previous case, we started our analysis with differences in the general population of patients with endometriosis compared to healthy volunteers. The obtained results are presented in Table 5.

On all analyzed T and B lymphocyte subpopulations, the expression of both PD-1 and PD-L1 was significantly higher in patients with endometriosis compared to healthy volunteers. The observed mean values were 2.92-fold higher for CD4+PD-1+, respectively; 6.43-fold for CD4+PD-L1+; 3.55-fold for CD8+PD-1+; 16.44-fold for CD8+PD-L1+; 4.82-fold for CD19+PD-1+; and 22.58-fold for CD19+PD-L1+. These trends were also maintained when analyzing serum concentrations of soluble forms of the tested molecules between patients. Thus, for sPD-1, the values were 1.93 times higher, while for sPD-L1, they were 1.65 times higher in patients with endometriosis. Additionally, we analyzed the concentration of tested molecules in the peritoneal fluid of patients with endometriosis, which showed an increase in the concentration of both sPD-1 (2.30-fold) and sPD-L1 (2.04-fold) about the values observed in the patient’s serum (Table 5).

**Figure 1:**
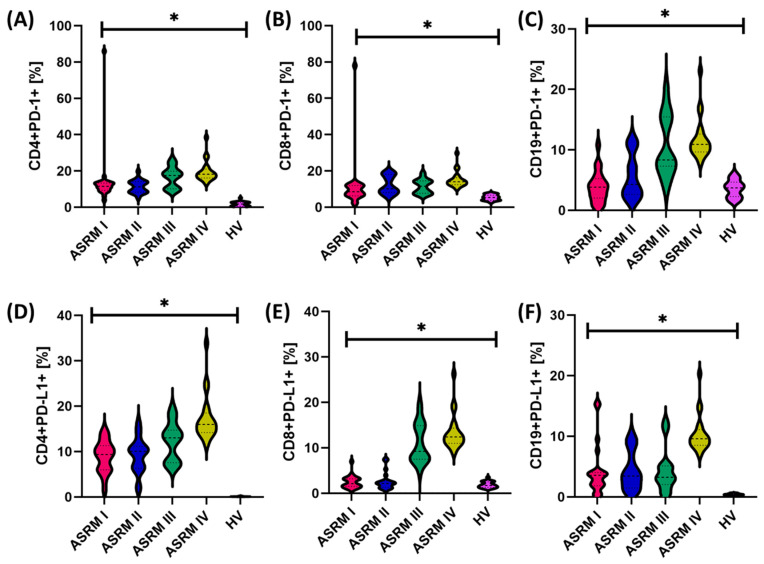
Graphical presentation of changes in the percentages of the tested particles on individual subpopulations of T lymphocytes (**A**,**B**,**D**,**E**) and B lymphocytes (**C**,**F**), taking into account the stages of endometriosis. The lines on the graph represent the first, second, and third quartiles, symbol * means statistically significant results.

In further analyses, we focused again on looking for differences between individual stages of patients with endometriosis. The obtained results are presented in Table 6. Our observations show that with the development of endometriosis (understood as the stage of advancement), the percentage of tested molecules on all tested subpopulations of T and B lymphocytes also increases. In the case of CD4+ T lymphocytes, significant differences were found between all stages t; for CD19+PD-1+, between I and III, I and IV, II and III, II and IV, and III and IV; and for CD19+PD-L1+, between I and IV, II and IV, and III and IV (Table 6) (Figure 1).

**Figure 2:**
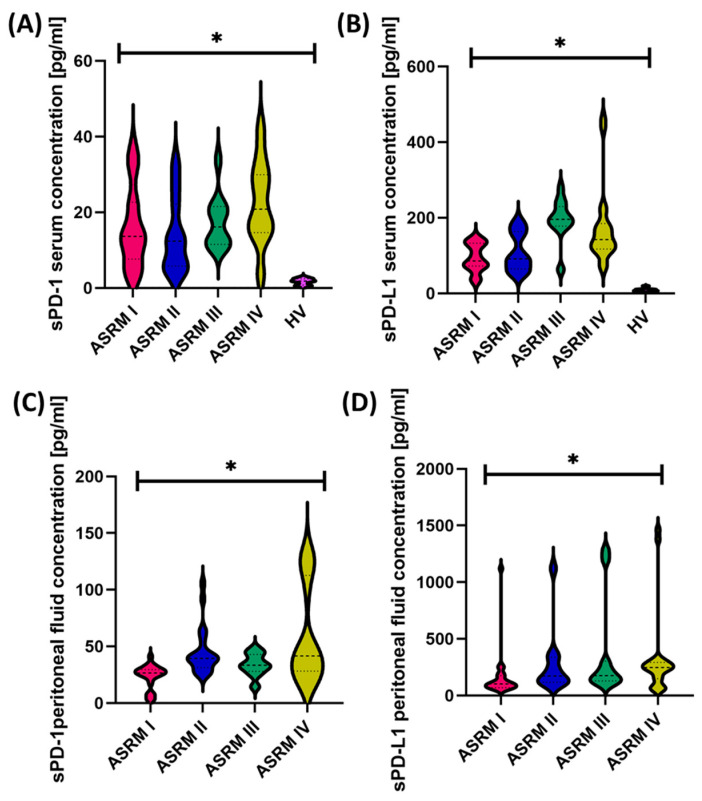
Graphical representation of changes in the concentration of tested molecules in serum (**A**,**B**) and peritoneal fluid (**C**,**D**), taking into account the stages of endometriosis. The lines on the graph represent the first, second, and third quartiles, symbol * means statistically significant results.

When analyzing the concentration of tested molecules in the serum of individual patients, we showed similar trends: the most significant differences were in the levels of sPD-1 between patients I and IV and II and IV, and for sPD-L1, between all stages except I and II. In the case of the analysis of the amount of sPD-1 and sPD-L1 in the peritoneal fluid, significant differences concerned the following groups of patients: I and II; I and III; and I and IV. Additionally, here, we also observed higher average concentration values of the tested molecules than in serum (Table 6) (Figure 2).

### 2.3. Correlation and ROC Curve Analysis

In the next stage, we decided to analyze the obtained research results in terms of potential correlations and the possibility of using the PD-1/PD-L1 pathway as biomarker molecules for the development and progression of endometriosis.

**Figure 3:**
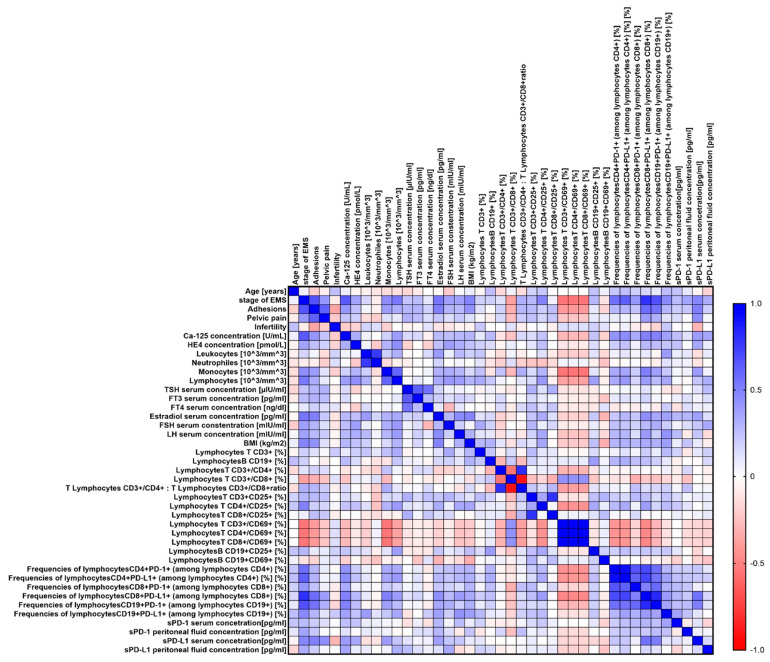
Graphical representation of Spearman’s rank correlation analysis for the entire population of endometriosis patients included in the study. Positive correlations are marked in blue, and negative correlations are marked in red.

**Figure 4:**
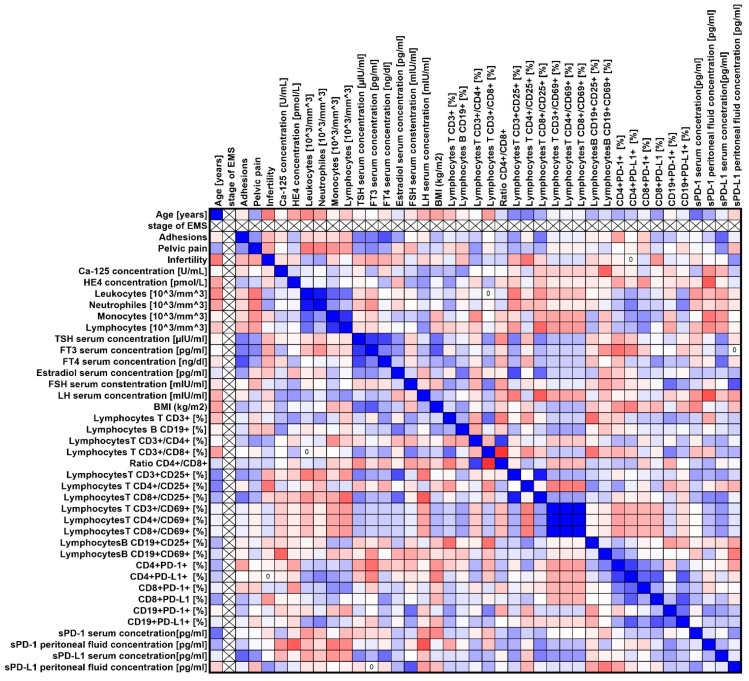
Graphical representation of Spearman’s rank correlation analysis for ARSM stage I patients with endometriosis included in the study. Positive correlations are marked in blue, and negative correlations are marked in red.

**Figure 5:**
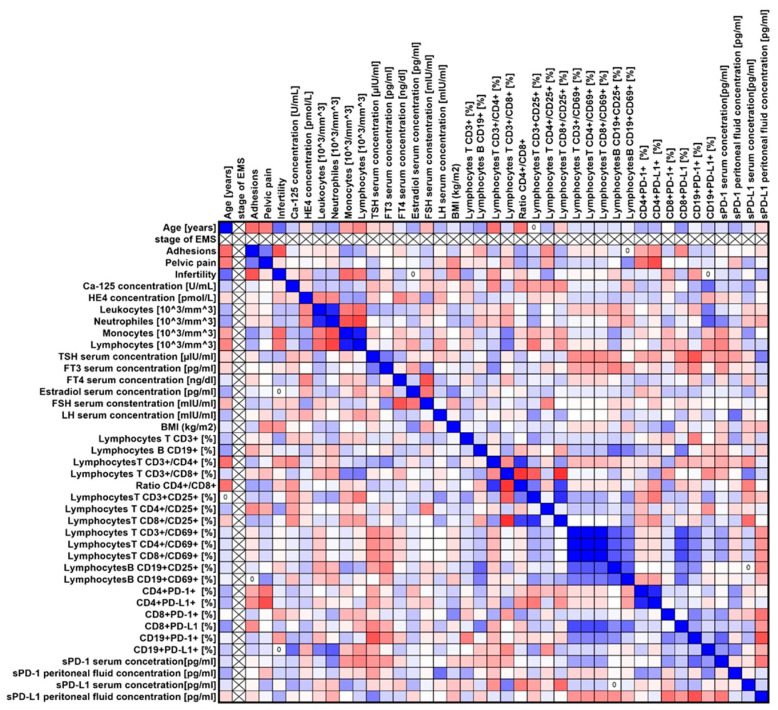
Graphical representation of Spearman’s rank correlation analysis for ARSM stage II endometriosis patients included in the study. Positive correlations are marked in blue, and negative correlations are marked in red.

**Figure 6:**
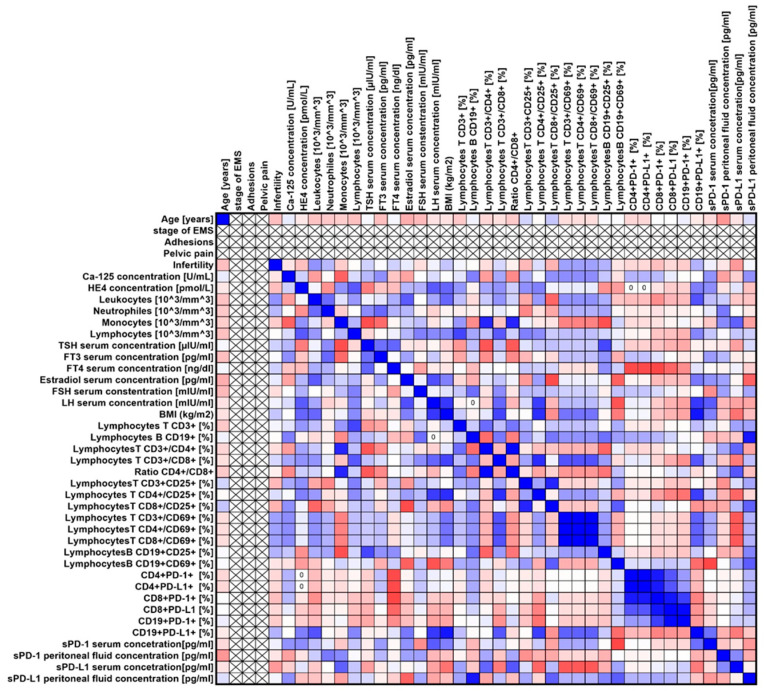
Graphical representation of Spearman’s rank correlation analysis for ARSM stage III patients with endometriosis included in the study. Positive correlations are marked in blue, and negative correlations are marked in red.

**Figure 7:**
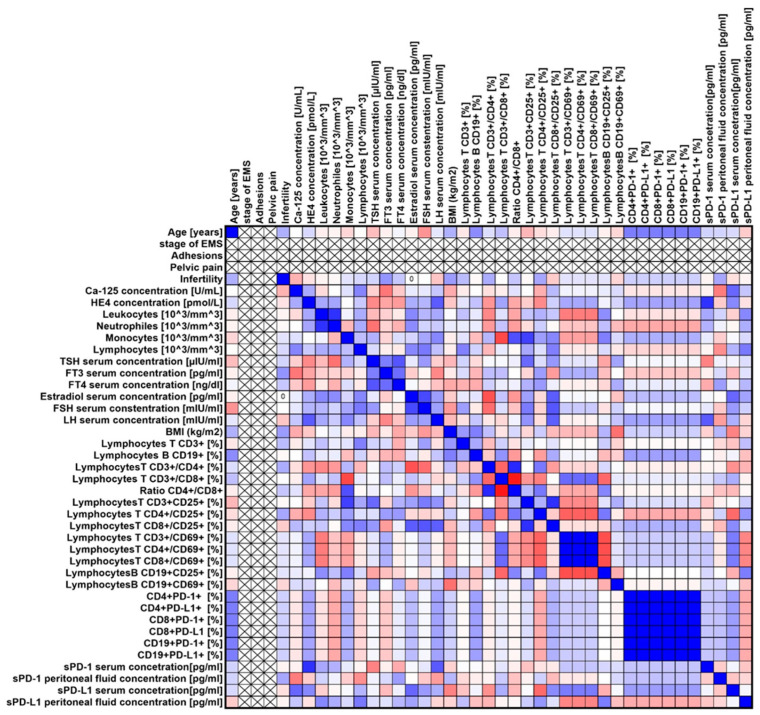
Graphical representation of Spearman’s rank correlation analysis for ARSM stage IV patients with endometriosis included in the study. Positive correlations are marked in blue, and negative correlations are marked in red.

Our analyses showed several significant correlations both for the entire group of patients with endometriosis (Figure 3) and in individual stages of advancement (Figure 4, Figure 5, Figure 6 and Figure 7).

**Figure 8:**
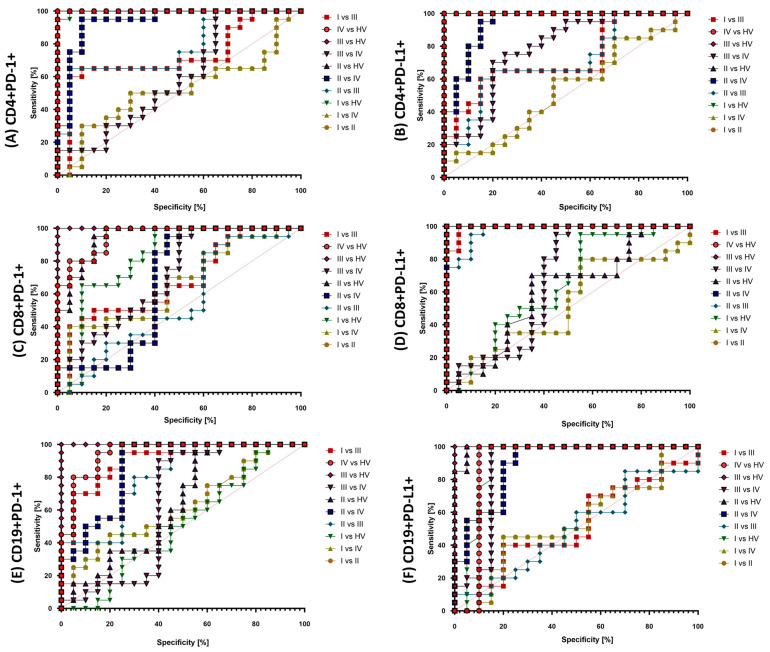
ROC curves for selected immunophenotyping parameters of the PD-1/PD-L1 pathway.

**Figure 9:**
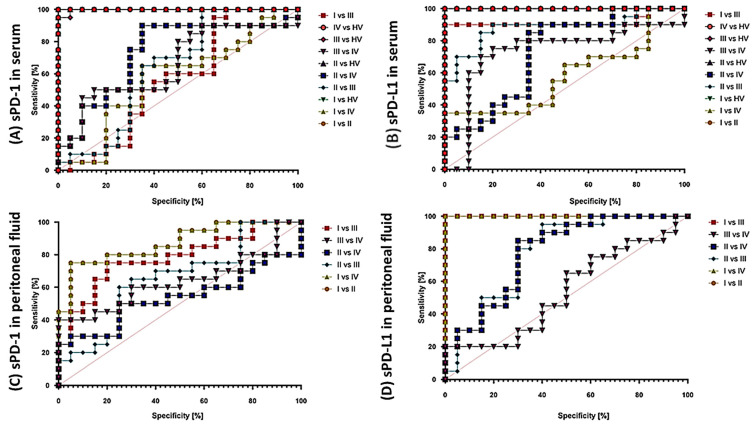
ROC curves for the assessment of sPD-1/sPD-L1 concentration in serum and peritoneal fluid.

The analysis of ROC curves proving the possibility of using the PD-1/PD-L1 pathway as potential molecules to monitor the development and progression of the disease also provided us with several important information. First of all, the use of these parameters may be a good solution when comparing the entire population of patients with endometriosis with healthy volunteers because in this aspect all analyzed parameters showed high sensitivity and specificity, both in immunophenotypic analyses and in serum concentration of molecules. The situation becomes a bit more complicated when comparing individual stages of endometriosis (Figure 8). The most sensitive parameter from our analysis may be CD8+PD-L1 between I and III and II and III, while in the case of serum concentrations, it is sPD-L1 between I and III, and in the case of the concentration in the peritoneal fluid, sPD-L1 between I and III and I and IV (Figure 9).

A detailed analysis of the evaluation of selected parameters of reliability, sensitivity, and specificity of diagnostic tests, carried out on a group of patients with endometriosis (taking into account the ASRM classification) and healthy volunteers, showed high sensitivity, specificity, and accuracy (ACC) in almost all tested parameters. These values ranged from 70 to 75%, and the accuracy (ACC) ranged from 72.5 to 75%. However, a comparative analysis of groups of endometriosis patients performed in the context of different stages of ASRM showed lower and more varied sensitivity and specificity of the tested parameters, ranging from 30% to 50% (ACC from 32.5% to 50%) (Supplementary Material Table S2). Moreover, due to the too small number of patients in the studied subgroups, the probability of using the analyzed molecules as potential biomarker molecules, useful diagnostically, showed only a small probability between individual types of ASRM and healthy volunteers, while in the case of comparisons between individual ASRM, the diagnostic test was insignificant or almost insignificant (Supplementary Material Table S2). Therefore, it is extremely important to conduct further studies on a much larger number of patients, which may contribute to the possibility of carrying out analyses allowing to increase the sensitivity, specificity, and probability of using the investigated PD-1/PD-L1 pathway as effective and useful molecules in the diagnosis of endometriosis. Despite obtaining significant research results, certain limitations make it impossible to draw final conclusions regarding the effectiveness of monitoring the PD-1/PD-L1 pathway in patients with endometriosis. One of the main limitations is the relatively small sample size of recruited patients. The study included newly diagnosed, untreated patients who were not using hormonal contraception or intrauterine devices, which presents a significant challenge given their widespread use in modern society. To increase the validity of our results, it is extremely important to expand the study cohort to include additional participants. Additionally, continued monitoring of these patients by our team will help validate our hypotheses and provide more reliable data on changes in PD-1/PD-L1 prevalence and concentrations over time, reflecting changes before and after initiation of treatment. The results presented in this study provide only preliminary insight into the role of the immune system, specifically the PD-1/PD-L1 pathway, in the development and progression of endometriosis. These findings constitute the basis for further interdisciplinary and comprehensive analyses. Although our team’s analyses of the use of PD-1/PD-L1 molecules as potential biomarker molecules showed only potential effectiveness in the ratio of patients with endometriosis to healthy volunteers, we did not observe the possibility of using them to differentiate ASRM in individual patients.

## 3. Discussion

One of the most important aspects of the immunological approach to endometriosis is the dysregulation of the immune response, which allows ectopic endometrial tissue to avoid destruction by the immune system, thereby promoting its implantation and growth. In this disease, interactions between T and B lymphocytes are observed, which can both enhance and inhibit pathological processes. T cells can stimulate B cells to produce antibodies, and cytokines produced by activated B cells can influence T cell differentiation and function. This dynamic interaction influences inflammation, immune tolerance, and the overall immune response in endometriosis [2,11,17,18,19]. Research has shown that there are statistically significant differences in the percentage of T and B lymphocytes between patients with endometriosis and healthy individuals. However, no significant differences have been observed in the immunophenotype of peripheral blood lymphocytes across different stages of endometriosis. Additionally, studies have indicated an increase in the activation of lymphocyte subpopulations in patients with endometriosis compared to healthy controls. Some differences have also been noted between various stages of endometriosis, suggesting that these cells play a role in the disease’s development and progression. The role of the PD-1/PD-L1 axis is not well-researched in the context of cancer, but its importance in the development and progression of endometriosis is becoming increasingly appreciated by researchers [20,21,22].

The expression of PD-1 on T cells in peripheral blood and PD-L1 on other immune cells and endometrial tissues is implicated in the immunosuppression observed in endometriosis. Evidence suggests that T cells with elevated PD-1 expression have diminished cytotoxic capacity against ectopic endometrial cells, facilitating immune evasion. Furthermore, increased PD-L1 expression on ectopic endometrial tissue enhances this evasion by creating an immune-tolerant environment.

Studies indicate that ectopic endometrial tissue exhibits heightened PD-L1 expression, which may serve as a defense mechanism against immune attacks. This overexpression inhibits T cell activation within ectopic tissues, reducing their cytotoxic response and thereby decreasing the elimination of pathological tissue, allowing for its continued proliferation [13,14,15,23].

In our studies, we showed that the percentage of lymphocytes expressing positive PD-1/PD-L1 was not only significantly higher in patients with endometriosis but also changed with the progression of the disease (endometriosis stage). These results are extremely interesting because they were conducted on newly diagnosed patients who were not undergoing treatment (including bridging treatment using contraception). This, therefore, reflects, at least to a small extent, the changes that occur at individual stages of endometriosis development. Hormone treatment is commonly used as a bridge therapy in the treatment of endometriosis. Its goal is to alleviate symptoms such as pain and discomfort and stop the progression of the disease. Hormone bridging therapy works by regulating hormone levels in the body, which helps reduce or eliminate ectopic endometrial tissue that responds to the menstrual cycle. The main types of hormonal treatments for endometriosis include oral contraceptives (containing a combination of estrogens and progestogens, which stabilize hormone levels and may reduce the bleeding and pain associated with endometriosis); GnRH agonists (gonadoliberins) (reducing the production of estrogen by the ovaries, which leads to reduced growth of endometrial tissue) or GnRH agonists (often used for short periods due to their potential side effects such as bone loss); and aromatase inhibitors (reducing the production estrogens in adipose tissues, which may be beneficial in the treatment of endometriosis resistant to other forms of hormonal therapy). Hormone therapy as a bridge therapy is used before elective surgery or as long-term management of endometriosis symptoms. It may also help reduce the risk of recurrence after surgery. The therapy must be tailored to the individual needs of the patient, taking into account her symptoms, fertility plans, and any contraindications to the use of specific medications. In this publication, we present the results regarding patients newly diagnosed and previously untreated with bridging therapies using hormonal therapies, which could influence the obtained research results. In the future, we also plan to research the impact of hormonal treatment on the development and progression of endometriosis [24,25,26].

Determining whether the PD-1/PD-L1 changes in T and B lymphocytes observed by our team are an epiphenomenon or a causative factor of endometriosis remains challenging. Immune system disorders, such as altered expression of PD-1 and PD-L1 on lymphocytes, might be a consequence of the disease rather than its direct cause. These aberrations may arise from the chronic inflammatory environment created by endometriotic lesions, leading to immune system dysregulation as the body attempts to manage persistent inflammation and tissue damage.

Conversely, there is evidence suggesting that immune dysfunction may play a crucial role in the pathogenesis of endometriosis. The immune system’s inability to effectively recognize and eliminate ectopic endometrial cells could contribute to the disease’s development and progression. Abnormal expression of immune checkpoints such as PD-1 and PD-L1 may impair the cytotoxic activity of T and B lymphocytes, allowing endometrial cells to evade immune surveillance and proliferate outside the uterine cavity.

Further longitudinal and mechanistic studies are required to ascertain whether these immune system abnormalities are a cause or consequence of endometriosis. These studies should investigate the temporal relationship between immune dysfunction and the development of endometriotic lesions, as well as the potential therapeutic benefits of targeting immune checkpoints in the treatment of endometriosis [12].

### 3.1. Limitations of the Conducted Study

While the preliminary results presented by our team are promising and were conducted on a specific group of newly diagnosed and previously untreated patients, our study has certain limitations. Primarily, the number of patients recruited for the study was limited. Due to the criteria adopted for the inclusion and exclusion of patients, which also pertain to the use of hormonal treatment, the number of participants is relatively small despite an extended recruitment period. We are striving to expand the research group, aiming to conduct analyses on a larger cohort. This would enable us to observe specific trends in the immunopathogenesis of endometriosis. Additionally, an important limitation is the one-time measurement of the immunological parameters at the time of recruitment. We hope that the patients will remain in close contact with our team, allowing us to monitor the progression of the disease and implement appropriate treatments, including hormonal therapy. This would facilitate longitudinal analyses of the measured parameters as the disease progresses, enhancing our understanding and treatment strategies. We acknowledge that the preliminary analyses presented by our team address only a small aspect of the changes occurring in the course of endometriosis. Therefore, we aim to broaden the spectrum of immunological parameters analyzed in future studies. This expansion will provide a more comprehensive understanding of the immune system’s role in the development and progression of this significant disease in the modern world.

### 3.2. Prospects for Research and Treatment of Endometriosis

Endometriosis, although not classified as a malignant tumor, exhibits structural similarities to malignant neoplasms due to its complex composition, including stromal, epithelial, and vascular elements, as well as genetic alterations. The incidence of abnormal (ectopic) cells in endometriosis-affected endometrium is significantly elevated compared to normal endometrial tissue. While prior studies have not indicated a heightened risk of malignant transformation in endometriosis patients, they have identified an increased incidence of ovarian cancer, endometrial cancer, non-Hodgkin’s lymphoma, colorectal cancer, thyroid cancer, and melanoma within this population [27,28,29,30,31,32,33].

Chronic inflammation is a pivotal factor in the pathogenesis of endometriosis. Proinflammatory cytokines produced at inflammatory sites can induce PD-L1 expression on endometrial cells and lymphocytes infiltrating endometriotic lesions. This induction may exacerbate local immunosuppression, sustain inflammation, and facilitate the proliferation and invasion of ectopic endometrial cells [19]. Elucidating the role of the PD-1/PD-L1 pathway in endometriosis opens new avenues for targeted therapeutic interventions. Inhibitors of PD-1 and PD-L1, which are currently utilized in oncology, hold the potential for modulating the immune response in endometriosis [31,32]. Therapeutic strategies aimed at downregulating PD-L1 expression or blocking the PD-1/PD-L1 interaction could enhance the immune system’s efficacy against ectopic endometrial tissue, thereby introducing novel treatment options for endometriosis management.

A critical component of advancing endometriosis research involves the education of both the public and healthcare professionals about the disease’s etiology and therapeutic approaches. Enhanced awareness of endometriosis symptoms, such as chronic pelvic pain, dysmenorrhea, and infertility, can lead to earlier diagnosis and intervention, thereby improving patient outcomes and preventing disease progression. Furthermore, public education fosters greater understanding and empathy towards those affected by endometriosis. An informed public is more likely to advocate for policies and initiatives that improve diagnostic and therapeutic access, including funding for research, the establishment of specialized medical centers, and the training of medical professionals [33].

## 4. Materials and Methods

### 4.1. Patients Included in the Study

The study group included 80 patients diagnosed with endometriosis based on ASRM guidelines and 20 healthy volunteers without reproductive system disorders (all recruited patients and healthy volunteers were female). Patients were subject to inclusion and exclusion criteria, which included the following:

- Histopathological and clinical confirmation of endometriosis;
- Age ≥ 18 years;
- Expected survival ≥ 12 months;
- No immunosuppressive treatment within a year before entering the study;
- No antibiotic therapy within three months before entering the study;
- Lack of use of contraceptives (hormonal contraception, intrauterine devices, etc.);
- Written consent to participate in the study.

Criteria for excluding endometriosis patients and healthy donors from the study include the following:

- Presence of leiomyomas and adenomyosis;
- Active viral, bacterial, or fungal infection;
- Diabetes or other metabolic and endocrine disorders;
- Severe allergies, chronic wounds, kidney diseases, intestinal diseases, cardiac diseases, pulmonary diseases, and/or use of a restrictive elimination diet;
- Condition after allotransplantation of hematopoietic cells or internal organs;
- Active malignancy or autoimmune disease;
- Period of pregnancy or lactation;
- Taking medications that affect the immune system or are undergoing clinical trials;
- Presence of cancer metastases within the central nervous system and/or mental illness.

Endometriosis was detected by a gynecologist with many years of experience in the diagnosis and treatment of this type of disease. First, ultrasound imaging of the pelvis (transvaginal and abdominal) and other body areas suspected of endometrial tissue presence was performed. In difficult cases, imaging using CT, MRI has also proven helpful in diagnosing endometriosis. However, the basic diagnosis was based on histopathological examination of diseased tissues collected during laparoscopy. In the case of patients from the control group, ultrasound imaging examinations of the pelvis (transvaginal and abdominal) and other areas of the body suspected of endometrial tissue were performed, which were negative. The test material was peripheral blood collected from the antecubital vein in a volume of 10 mL into test tubes containing EDTA (to perform immunophenotyping) (Sarstedt, Germany), 5 mL into test tubes with a coagulation activator (to determine the levels of tested molecules and hormones). Blood sampling in all recruited patients was performed in the first phase of the cycle due to the possibility of a better assessment of the uterine mucosa (between cycle days 5 and 8).

For the peritoneal fluid (PF) samples, these were also collected during the early proliferative phase, corresponding to the same range of cycle days, between day 5 and day 8 of the menstrual cycle. In the case of patients from the study group, peritoneal fluid was also collected. The average age of the recruited patients was 34.6 ± 6.18 years for the study group and 36.6 ± 6.69 years for the healthy volunteers. The BMI values for the research group’s patients were 23.6 ± 2.15, and for the healthy volunteers, they were 22.8 ± 1.84. The demographic data for the research group indicated that 43 patients resided in a city with a population of 200,000 to 500,000 inhabitants, 10 patients lived in a city with 100,000 to 200,000 inhabitants, 13 patients lived in a city with 50,000 to 100,000 residents, and 14 patients were from rural areas. In comparison, the control group comprised 7 patients from a city with 200,000 to 500,000 inhabitants, 4 patients from a city with 100,000 to 200,000 inhabitants, 6 patients from a city with 50,000 to 100,000 inhabitants, and 3 patients from rural areas. In 53 (66.25%) patients with endometriosis, adhesions were observed (they form and cause scarring in the ovaries, fallopian tubes, uterus, small intestine, and side walls of the pelvis, between the intestine, rectum, and rectovaginal septum). Additionally, 83.75% of recruited patients complained of pain in the pelvic area, and 52.5% were diagnosed with infertility.

All patients included in the study were not previously treated or used hormonal contraception and/or intrauterine devices. Additionally, all recruited patients and healthy volunteers were not subject to anti-inflammatory treatment (using non-steroidal anti-inflammatory drugs, corticosteroids, or biological treatment) before starting the study.

### 4.2. Immunophenotyping

**Figure 10:**
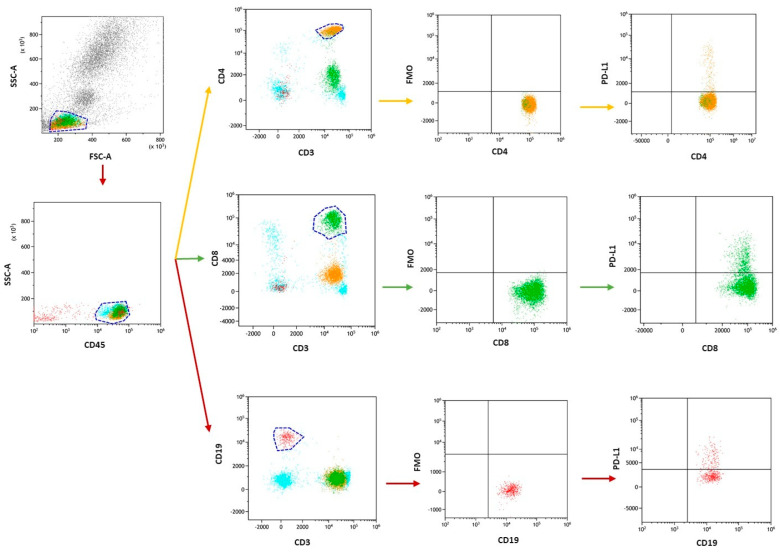
Exemplary analysis of the cells’ immunophenotypes and the determination of the percentage of positive PD-1/PD-L1 expression. In the figure, an example of PD-L1 expression on the CD4+CD3+ subpopulation is marked in orange, CD8+CD3+ subpopulation in green, and CD19+CD3− subpopulation in red, CD45+ subpopulation in blue. Method of reading PD-L1 using the FMO control.

Low cytometry was used to analyze the immunophenotype of lymphocytes in peripheral blood. A whole blood sample was collected and treated with a panel of human monoclonal antibodies, including anti-CD45 AF700, anti-CD3 PerCp, anti-CD4 BV421, anti-CD8 BV605, anti-CD19 FITC, anti-CD56 BV650, anti-CD56 BV650, CD16 BV650, anti-PD-1 APC, and anti-PD-L1 PE. All antibodies were from BioLegend (San Diego, CA, USA). Any remaining red blood cells were removed using BD FACS™ Lysing Solution 10× Concentrate (BD, Becton, Dickinson, NJ, USA) prepared according to the manufacturer’s instructions. After lysis, cells were washed using BD Pharmingen™ Stain Buffer (BSA) (BD, Becton, Dickinson, NJ, USA), and then cells were assessed using a flow cytometer, CytoFLEX LX (Beckman Coulter, Indianapolis, IN, USA). The number of CD45+ gate events was 15,000. Data were analyzed using Kaluza Analysis software v. 2.1, as shown in Figure 10. To ensure accuracy, the CytoFLEX LX flow cytometer was subjected to daily quality control using CytoFLEX Ready to Use Daily QC Fluorphers from Beckman Coulter.

### 4.3. Determination of the Concentration of Soluble Forms of PD-1/PD-L1 in Serum and Peritoneal Fluid

The commercially available Human PD-1 ELISA Kit (range: 25–1600 pg/mL; sensitivity = 9.6 pg/mL) and Human PD-L1 ELISA were used to determine the concentration of soluble forms of PD-1/PD-L1 in serum and peritoneal fluid. Kit (range: 7.81–500 pg/mL; sensitivity = 3.75 pg/mL) was from abcam (Cambridge, UK). The tests were performed following the manufacturer’s instructions, using the Victor 3.0 reader with Workout 2.0 software.

### 4.4. Statistical Analyses

Statistical analyses of the collected data were conducted using Tibco Statistica 13.3 software, based in Palo Alto, CA, USA. The Shapiro–Wilk test was employed to evaluate the normality of the data distribution. Group differences were examined with the Kruskal–Wallis test, followed by Dunn’s post hoc test, with *p*-values adjusted for multiple comparisons via the Bonferroni method. Spearman’s correlation coefficients were calculated to investigate the relationships between variable pairs. ROC curves were utilized to determine the diagnostic efficacy of laboratory tests regarding patient-related parameters. Additionally, data visualizations were created using GraphPad Prism Software version 9.4.1, located in San Diego, CA, USA.

## 5. Conclusions

Studying the role of PD-1/PD-L1 in the context of T and B lymphocytes may provide valuable information not only about the immunological mechanisms of endometriosis but also about the potential possibilities of immunotherapeutic interventions that could revolutionize the approach to the treatment of this difficult-to-control disease.

Despite increasing knowledge about the role of the PD-1/PD-L1 axis in endometriosis, further research is still needed to thoroughly understand the mechanisms and apply this information to clinical practice. These studies should focus on further examining the role of PD-1 and PD-L1 in different T and B cell subpopulations and their interactions with other components of the immune system in the context of endometriosis.

## Figures and Tables

**Table 1:** Analysis of selected morphology and biochemistry results of peripheral blood in patients with endometriosis in comparison to healthy volunteers.

Parameters	Study Group (*n* = 80)		Healthy Volunteers (*n* = 20)		*p*-Value
	Mean ± SD	Median (Range)	Mean ± SD	Median (Range)	
Leukocytes [10^3^/mm^3^]	8.71 ± 1.61	8.74 (5.51–12.01)	9.23 ± 5.23	9.02 (2.50–21.73)	0.949
Neutrophiles [10^3^/mm^3^]	5.23 ± 1.31	5.61 (2.08–7.91)	3.05 ± 0.90	3.76 (1.69–5.18)	0.000 *
Monocytes [10^3^/mm^3^]	0.58 ± 0.18	0.59 (0.24–0.96)	0.58 ± 0.18	0.59 (0.24–0.96)	0.000 *
Lymphocytes [10^3^/mm^3^]	4.32 ± 1.00	3.94 (2.71–6.03)	2.92 ± 1.32	2.73 (1.20–7.54)	0.000 *
Ca-125 concentration [U/mL]	42.37 ± 21.31	35.36 (7.67–107.06)	4.52 ± 2.71	3.50 (1.30–10.56)	0.000 *
HE4 concentration [pmol/L]	42.06 ± 10.48	40.85 (26.00–76.71)	NA	NA	NA
TSH serum concentration [µIU/mL]	1.41 ± 0.63	1.43 (0.33–2.76)	1.43 ± 0.67	1.50 (0.44–2.55)	0.907
FT3 serum concentration [pg/mL]	3.19 ± 0.58	3.12 (2.01–4.40)	3.13 ± 0.56	3.12 (2.02–4.12)	0.908
FT4 serum concentration [ng/dL]	1.34 ± 0.24	1.34 (0.92–1.79)	1.30 ± 0.22	1.30 (0.93–1.67)	0.691
Estradiol serum concentration [pg/mL]	56.87 ± 27.10	49.65 (14.50–120.16)	56.23 ± 26.74	44.95 (25.60–112.30)	0.502
FSH serum constentration [mIU/mL]	6.35 ± 1.83	6.10 (2.20–10.39)	6.17 ± 1.54	6.00 (3.50–8.50)	0.755
LH serum concentration [mIU/mL]	8.36 ± 3.55	7.90 (3.60–16.96)	5.96 ± 1.78	5.75 (3.20–10.50)	0.729

NA—not applicable; HE4—human epididymis protein 4; TSH—thyroid-stimulating hormone; FT3—free triiodothyronine; FT4—free thyroxine; FSH—follicle-stimulating hormone; LH—luteinizing hormone; and * statistically significant results.

**Table 2:** Immunophenotyping analyses of peripheral blood of patients with endometriosis compared to healthy volunteers.

Parameters	Study Group (*n* = 80)		Healthy Volunteers (*n* = 20)		*p*-Value
	Mean ± SD	Median (Range)	Mean ± SD	Median (Range)	
Lymphocytes T CD3+ [%]	72.09 ± 5.16	72.92 (61.31–83.50)	68.26 ± 3.74	68.08 (60.63–74.49)	0.000 *
Lymphocytes B CD19+ [%]	10.50 ± 3.07	9.86 (6.12–17.50)	11.25 ± 2.44	11.40 (6.04–16.90)	0.000 *
Lymphocytes T CD3+/CD4+ [%]	43.74 ± 7.94	44.37 (26.13–69.38)	44.46 ± 2.44	44.16 (40.71–48.84)	0.952
Lymphocytes T CD3+/CD8+ [%]	28.39 ± 6.80	28.38 (16.25–43.43)	34.36 ± 3.20	34.74 (29.33–39.60)	0.000 *
Lymphocytes CD3+/CD4+: T Lymphocytes CD3+/CD8+ratio	1.72 ± 0.67	1.67 (0.67–4.11)	1.31 ± 0.15	1.29 (1.03–1.57)	0.000 *
Lymphocytes T CD3+CD25+ [%]	28.99 ± 8.33	26.96 (10.86–56.29)	7.60 ± 2.69	8.03 (1.08–11.13)	0.000 *
Lymphocytes T CD4+/CD25+ [%]	14.93 ± 5.88	14.99 (0.82–29.48)	5.66 ± 2.40	6.35 (0.95–8.83)	0.000 *
Lymphocytes T CD8+/CD25+ [%]	14.06 ± 8.53	12.12 (2.23–35.41)	1.94 ± 1.11	1.63 (0.13–5.11)	0.000 *
Lymphocytes T CD3+/CD69+ [%]	13.86 ± 8.50	13.84 (1.21–33.66)	3.38 ± 1.66	3.36 (0.52–6.89)	0.004 *
Lymphocytes T CD4+/CD69+ [%]	8.91 ± 5.47	8.90 (0.78–21.64)	2.20 ± 1.00	2.30 (0.18–3.48)	0.000 *
Lymphocytes T CD8+/CD69+ [%]	4.95 ± 3.04	4.94 (0.43–12.02)	1.18 ± 1.19	0.70 (0.02–3.87)	0.000 *
Lymphocytes B CD19+CD25+ [%]	3.63 ± 1.84	3.10 (0.55–8.14)	1.77 ± 1.29	1.81 (0.06–5.12)	0.046 *
Lymphocytes B CD19+CD69+ [%]	2.21 ± 0.99	2.01 (0.06–6.65)	0.12 ± 0.06	0.09 (0.06–0.25)	0.003 *

CD—cluster of differentiation; * statistically significant results.

**Table 3:** Values of selected peripheral blood morphology and biochemistry parameters in patients with endometriosis, with particular emphasis on the stage of the disease.

Parameters	Stage I	Stage II	Stage III	Stage IV	Healthy Volunteers (V)	*p*-Value										
	Median (Range)	Median (Range)	Median (Range)	Median (Range)	Median (Range)	I vs. II	I vs. III	I vs. IV	I vs. V	II vs. III	II vs. IV	II vs. V	III vs. IV	III vs. V	IV vs. V	All
Leukocytes [10^3^/mm^3^]	8.15 (6.16–11.15)	8.26 (6.06–10.40)	8.92 (5.51–10.93)	10.10 (5.83–12.01)	9.02 (2.50–21.73)	0.678	0.659	0.289	0.925	0.289	0.102	0.779	0.242	0.947	0.799	0.289
Neutrophiles [10^3^/mm^3^]	5.78 (2.62–7.48)	5.52 (2.08–7.91)	5.29 (4.14–7.43)	5.63 (2.57–7.67)	3.76 (1.69–5.18)	0.289	0.820	0.265	0.000 *	0.314	0.820	0.000 *	0.301	0.000 *	0.000 *	0.000 *
Monocytes [10^3^/mm^3^]	0.49 (0.24–0.78)	0.47 (0.29–0.63)	0.71 (0,32–0.96)	0.69 (0.42–0.86)	0.59 (0.24–0.96)	0.495	0.007 *	0.003 *	0.000 *	0.001 *	0.000 *	0.000 *	0.718	0.000 *	0.000 *	0.000 *
Lymphocytes [10^3^/mm^3^]	2.14 (1.20–3.94)	2.29 (1.30–3.14)	2.64 (1.32–7.54)	3.61 (2.39–6.91)	3.94 (2.71–6.03)	0.799	0.068	0.000 *	0.000 *	0.097	0.000 *	0.000 *	0.014 *	0.001 *	0.221	0.000 *
Ca-125 concentration [U/mL]	18.43 (7.67–47.89)	34.10 (18.29–78.82)	48.18 (15.56–71.20)	53.35 (32.16–107.06)	3.50 (1.30–10.56)	0.000 *	0.000 *	0.000 *	0.000 *	0.383	0.004 *	0.000 *	0.018 *	0.000 *	0.000 *	0.000 *
HE4 concentration [pmol/L]	38.15 (27.00–60.30)	39.65 (26.00–65.00)	41.10 (27.30–76.71)	44.41 (31.00–62.86)	NA	0.355	0.033 *	0.000 *	NA	0.659	0.201	NA	0.201	NA	NA	NA
TSH serum concentration [µIU/mL]	1.09 (0.33–2.34)	1.16 (0.35–2.57)	1.62 (0.68–2.76)	1.78 (0.44–2.49)	1.50 (0.44–2.55)	0.398	0.018 *	0.121	0.398	0.157	0.341	0.398	0.718	0.398	0.398	0.02 *
FT3 serum concentration [pg/mL]	2.96 (2.12–4.34)	3.06 (2.01–3.93)	3.33 (2.46–4.40)	3.47 (2.74–4.25)	3.12 (2.02–4.12)	0.841	0.157	0.068	0.000 *	0.149	0.028 *	0.000 *	0.383	0.000 *	0.000 *	0.000 *
FT4 serum concentration [ng/dL]	1.37 (0.94–1.65)	1.31 (0.96–1.67)	1.31 (0.92–1.79)	1.39 (0.96–1.77)	1.30 (0.93–1.67)	0.495	0.968	0.429	0.678	0.583	0.192	0.602	0.512	0.678	0.779	0.192
Estradiol serum concentration [pg/mL]	34.50 (14.50–56.30)	46.45 (28.20–60.20)	65.67 (30.80–120.16)	76.74 (38.20–111.50)	44.95 (25.60–112.30)	0.091	0.000 *	0.000 *	0.000 *	0.040 *	0.000 *	0.000 *	0.820	0.000	0.000 *	0.000 *
FSH serum constentration [mIU/mL]	5.10 (2.20–7.80)	6.10 (3.80–9.50)	6.73 (4.20–9.52)	7.71 (3.90–10.39)	6.00 (3.50–8.50)	0.127	0.028 *	0.000 *	0.000 *	0.495	0.102	0.000 *	0.398	0.000 *	0.000 *	0.000 *
LH serum concentration [mIU/mL]	7.45 (3.90–13.20)	6.00 (3.80–14.60)	9.57 (3.90–15.80)	10.20 (3.60–16.96)	5.75 (3.20–10.50)	0.221	0.072	0.006 *	0.000 *	0.006 *	0.000 *	0.000 *	0.355	0.000 *	0.000 *	0.000 *

NA—not applicable; HE4—human epididymis protein 4; TSH—thyroid-stimulating hormone; FT3—free triiodothyronine; FT4—free thyroxine; FSH—follicle-stimulating hormone; LH—luteinizing hormone; and * statistically significant results.

**Table 4:** Values of selected peripheral blood immunophenotyping parameters in patients with endometriosis with particular emphasis on the stage of the disease.

Parameters	Stage I	Stage II	Stage III	Stage IV	Healthy Volunteers (V)	*p*-Value										
	Median (Range)	Median (Range)	Median (Range)	Median (Range)	Median (Range)	I vs. II	I vs. III	I vs. IV	I vs. V	II vs. III	II vs. IV	II vs. V	III vs. IV	III vs. V	IV vs. V	All
Lymphocytes T CD3+ [%]	74.35 (64.20–78.54)	69.86 (62.80–75.72)	70.55 (61.88–81.51)	75.60 (61.31–83.50)	68.08 (60.63–74.49)	0.127	0.583	0.086	0.000 *	0.565	0.007 *	0.000 *	0.063	0.000 *	0.000 *	0.000 *
Lymphocytes B CD19+ [%]	9.08 (6.14–16.84)	9.76 (6.30–17.50)	9.59 (6.12–16.32)	11.58 (7.69–17.33)	11.40 (6.04–16.90)	0.398	0.989	0.038 *	0.000 *	0.529	0.277	0.000 *	0.043 *	0.000 *	0.000 *	0.000 *
Lymphocytes T CD3+/CD4+ [%]	43.99 (32.73–48.81)	44.55 (26.13–54.97)	44.66 (26.62–54.61)	45.95 (27.34–69.38)	44.16 (40.71–48.84)	0.529	0.512	0.327	0.000 *	0.862	0.512	0.000 *	0.478	0.000 *	0.000 *	0.000 *
Lymphocytes T CD3+/CD8+ [%]	31.93 (26.32–40.75)	22.58 (20.12–42.90)	27.12 (24.56–39.60)	23.48 (16.25–43.43)	34.74 (29.33–39.60)	0.007 *	0.001 *	0.001 *	0.000 *	0.108	0.134	0.000 *	0.192	0.000 *	0.000 *	0.000 *
Lymphocytes CD3+/CD4+: T Lymphocytes CD3+/CD8+ratio	1.29 (0.98–1.80)	1.96 (0.68–2.40)	1.78 (0.85–2.10)	1.96 (0.67–4.11)	1.29 (1.03–1.57)	0.020 *	0.014 *	0.003 *	0.000 *	0.091	0.355	0.000 *	0.142	0.000 *	0.000 *	0.000 *
Lymphocytes T CD3+CD25+ [%]	26.56 (17.90–48.67)	25.96 (10.86–38.79)	25.09 (16.88–43.61)	30.89 (23.37–56.29)	8.03 (1.08–11.13)	0.718	0.904	0.007 *	0.000 *	0.862	0.006 *	0.000 *	0.004 *	0.000 *	0.565	0.000 *
Lymphocytes T CD4+/CD25+ [%]	9.61 (0.82–29.48)	14.27 (1.97–21.60)	14.45 (11.84–25.30)	18.79 (11.08–27.45)	6.35 (0.95–8.83)	0.314	0.046 *	0.000 *	0.000 *	0.369	0.000 *	0.000 *	0.011 *	0.000 *	0.000 *	0.000 *
Lymphocytes T CD8+/CD25+ [%]	10.93 (2.23–31.72)	16.60 (2.75–35.41)	11.12 (4.06–20.45)	12.66 (5.29–34.98)	1.63 (0.13–5.11)	1.000	0.698	0.495	0.091	0.512	0.529	0.121	0.086	0.023 *	0.001 *	0.000 *
Lymphocytes T CD3+/CD69+ [%]	19.62 (2.84–33.66)	19.71 (6.56–27.30)	8.86 (1.52–18.47)	5.11 (1.21–22.38)	3.36 (0.52–6.89)	0.862	0.000 *	0.000 *	0.000 *	0.000 *	0.000 *	0.000 *	0.242	0.012 *	0.659	0.000 *
Lymphocytes T CD4+/CD69+ [%]	12.62 (1.83–21.64)	12.67 (4.22–17.55)	5.69 (0.98–11.88)	3.29 (0.78–14.38)	2.30 (0.18–3.48)	0.862	0.000 *	0.000 *	0.000 *	0.000 *	0.000 *	0.000 *	0.242	0.000 *	0.035 *	0.000 *
Lymphocytes T CD8+/CD69+ [%]	7.01 (1.01–12.02)	7.04 (2.34–9.75)	3.17 (0.54–6.59)	1.83 (0.43–7.99)	0.70 (0.02–3.87)	0.883	0.000 *	0.000 *	0.000 *	0.000 *	0.000 *	0.000 *	0.242	0.925	0.142	0.000 *
Lymphocytes B CD19+CD25+ [%]	3.10 (1.35–5.13)	3.12 (0.55–7.38)	2.38 (1.48–4.64)	5.92 (1.99–8.14)	1.81 (0.06–5.12)	0.904	0.114	0.000 *	0.026 *	0.052	0.002 *	0.028 *	0.000 *	0.512	0.000 *	0.000 *
Lymphocytes B CD19+CD69+ [%]	2.11 (1.06–4.59)	2.17 (1.44–6.65)	1.71 (1.26–2.41)	2.29 (0.06–3.45)	0.09 (0.06–0.25)	0.718	0.009 *	0.512	0.000 *	0.006 *	0.565	0.002 *	0.009 *	0.007 *	0.013 *	0.000 *

CD—cluster of differentiation; * statistically significant results.

**Table 5:** Results of the percentage of PD-1/PD-L1 occurrence on selected immune cell populations in patients with endometriosis compared to healthy volunteers.

Parameters	Study Group		Healthy Volunteers		*p*-Value
	Mean ± SD	Median (Range)	Mean ± SD	Median (Range)	
CD4+PD-1+ [%]	15.67 ± 9.76	13.89 (3.92–86.01)	5.35 ± 1.50	5.35 (2.65–7.69)	0.000 *
CD4+PD-L1+ [%]	11.96 ± 5.29	11.70 (1.43–33.87)	1.86 ± 0.68	1.71 (0.98–3.49)	0.000 *
CD8+PD-1+ [%]	12.80 ± 8.67	11.99 (1.90–77.98)	3.60 ± 1.42	3.71 (1.36–6.17)	0.000 *
CD8+PD-L1+ [%]	7.40 ± 5.77	6.97 (0.72–26.23)	0.45 ± 0.11	0.43 (0.31–0.67)	0.000 *
CD19+PD-1+ [%]	8.05 ± 4.97	7.70 (0.54–23.08)	1.67 ± 0.82	1.81 (0.37–3.01)	0.000 *
CD19+PD-L1+ [%]	5.87 ± 4.34	4.25 (0.13–20.31)	0.26 ± 0.22	0.20 (0.07–1.03)	0.000 *
sPD-1 serum concentration [pg/mL]	17.67 ± 10.00	14.49 (2.95–43.27)	9.12 ± 4.01	8.03 (4.63–19.82)	0.000 *
sPD-1 peritoneal fluid concentration [pg/mL]	40.70 ± 27.58	33.04 (2.74–133.67)	NA	NA	NA
sPD-L1 serum concentration [pg/mL]	143.14 ± 76.82	135.80 (30.53–463.03)	86.79 ± 40.16	79.34 (38.38–172.68)	0.000 *
sPD-L1 peritoneal fluid concentration [pg/mL]	292.30 ± 347.20	162.69 (60.20–1462.27)	NA	NA	NA

CD—cluster of differentiation; NA—not applicable; * statistically significant results.

**Table 6:** Analysis of the percentage of PD-1/PD-L1 occurrence on selected immune system cells with particular emphasis on the stage of endometriosis.

Parameters	Stage I	Stage II	Stage III	Stage IV	Healthy Volunteers (V)	*p*-Value										
	Median (Range)	Median (Range)	Median (Range)	Median (Range)	Median (Range)	I vs. II	I vs. III	I vs. IV	I vs. V	II vs. III	II vs. IV	II vs. V	III vs. IV	III vs. V	IV vs. V	All
CD4+PD-1+ [%]	11.58 (3.92–86.01)	11.27 (5.28–19.74)	17.58 (8.84–26.58)	18.18 (13.06–38.49)	5.35 (2.65–7.69)	0.841	0.015 *	0.000 *	0.000 *	0.002 *	0.000 *	0.000 *	0.314	0.000 *	0.000 *	0.000 *
CD4+PD-L1+ [%]	9.36 (1.43–14.76)	10.06 (1.70–16.51)	13.01 (6.54–19.67)	16.00 (11.49–33.87)	1.71 (0.98–3.49)	0.758	0.014 *	0.000 *	0.000 *	0.024 *	0.000 *	0.000 *	0.002 *	0.000 *	0.000 *	0.000 *
CD8+PD-1+ [%]	8.52 (1.90–77.98)	10.23 (5.52–20.10)	11.55 (6.29–19.12)	14.08 (10.11–29.81)	3.71 (1.36–6.17)	0.097	0.060	0.000 *	0.000 *	0.602	0.076	0.000 *	0.023 *	0.000 *	0.000 *	0.000 *
CD8+PD-L1+ [%]	2.12 (0.87–6.97)	2.16 (0.72–7.55)	5.14 (0.52–12.53)	12.39 (8.90–26.23)	0.43 (0.31–0.67)	0.820	0.000 *	0.000 *	0.127	0.000 *	0.000 *	0.265	0.072	0.000 *	0.000 *	0.000 *
CD19+PD-1+ [%]	3.83 (0.54–10.82)	4.29 (1.38–11.82)	9.23 (4.65–19.88)	10.90 (7.83–23.08)	1.81 (0.37–3.01)	0.192	0.000 *	0.000 *	0.947	0.001 *	0.000 *	0.142	0.149	0.000 *	0.000 *	0.000 *
CD19+PD-L1+ [%]	3.56 (0.14–15.50)	3.44 (0.59–10.40)	8.33 (3.44–20.68)	9.59 (6.89–20.31)	0.20 (0.07–1.03)	0.640	0.862	0.000 *	0.000 *	0.989	0.000 *	0.000 *	0.000 *	0.000 *	0.000 *	0.000 *
sPD-1 serum concentration [pg/mL]	13.62 (3.75–37.45)	12.37 (3.68–33.74)	16.13 (9.65–34.99)	20.81 (2.95–43.27)	8.03 (4.63–19.82)	0.445	0.512	0.043 *	0.000 *	0.142	0.011 *	0.000 *	0.086	0.000 *	0.000 *	0.000 *
sPD-1 peritoneal fluid concentration [pg/mL]	26.63 (2.74–40.99)	39.43 (25.36–105.73)	33.35 (13.91–48.79)	41.66 (18.19–133.67)	NA	0.000 *	0.002 *	0.001 *	NA	0.097	0.698	NA	0.142	NA	NA	NA
sPD-L1 serum concentration [pg/mL]	85.81 (30.53–147.24)	91.03 (49.10–192.20)	195.52 (60.91–284.87)	142.29 (70.53–463.03)	79.34 (38.38–172.68)	0.383	0.000 *	0.001 *	0.000 *	0.000 *	0.013 *	0.000 *	0.013 *	0.000 *	0.000 *	0.000 *
sPD-L1 peritoneal fluid concentration [pg/mL]	101.99 (62.49–1121.24)	172.54 (86.55–1141.10)	175.12 (90.60–1303.15)	247.67 (60.20–1462.27)	NA	0.002 *	0.002 *	0.013 *	NA	0.698	0.602	NA	0.529	NA	NA	0.000 *

NA—not applicable; * statistically significant results.

## Data Availability

The data presented in this publication are available from the first author upon written request.

## Supplementary Materials

The following supporting information can be downloaded at https://www.mdpi.com/article/10.3390/ijms25126775/s1.
