# Supplementary material for: The PD-1/PD-L1 Gateway: Peripheral Immune Regulation in the Pathogenesis of Endometriosis
Source: Int J Mol Sci. 2024 Jun 20;25(12):6775. doi: 10.3390/ijms25126775 (PMC11203925; doi:10.3390/ijms25126775)
Supplement: Supplementary file 1 [file ijms-25-06775-s001.zip › ijms-3015195-supplementary.pdf]

Supplementary Materials Table S1: ROC curves analysis

Parameters	CD4+PD-1+ [%]									
	I vs. II	I vs. III	I vs. IV	II vs. III	II vs. IV	III vs. IV	I vs. HV	II vs. HV	III vs. HV	IV vs. HV
Area	0.52	0.72	0.92	0.78	0.94	0.60	1.00	1.00	1.00	1.00
Std. Error	0.09	0.09	0.05	0.08	0.04	0.09	0.00	0.00	0.00	0.00
95% confidence interval	0.3341 to 0.7059	0.5544 to 0.8906	0.8166 to 1.000	0.6302 to 0.9248	0.8578 to 1.000	0.4145 to 0.7755	0.9893 to 1.000	1.000 to 1.000	1.000 to 1.000	1.000 to 1.000
P value	0.83	0.02	<0.0001	0.00	<0.0001	0.30	<0.0001	<0.0001	<0.0001	<0.0001
	CD4+PD-L1+ [%]									
	I vs. II	I vs. III	I vs. IV	II vs. III	II vs. IV	III vs. IV	I vs. HV	II vs. HV	III vs. HV	IV vs. HV
Area	0.53	0.73	0.97	0.71	0.94	0.78	1.00	1.00	1.00	1.00
Std. Error	0.09	0.08	0.02	0.08	0.04	0.07	0.00	0.00	0.00	0.00
95% confidence interval	0.3468 to 0.7107	0.5645 to 0.8855	0.9181 to 1.000	0.5437 to 0.8713	0.8620 to 1.000	0.6371 to 0.9279	1.000 to 1.000	1.000 to 1.000	1.000 to 1.000	1.000 to 1.000
P value	0.76	0.01	<0.0001	0.02	<0.0001	0.00	<0.0001	<0.0001	<0.0001	<0.0001
	CD8+PD-1+ [%]									
	I vs. II	I vs. III	I vs. IV	II vs. III	II vs. IV	III vs. IV	I vs. HV	II vs. HV	III vs. HV	IV vs. HV
Area	0.66	0.68	0.88	0.55	0.67	0.71	0.82	0.95	0.96	1.00
Std. Error	0.09	0.09	0.06	0.09	0.09	0.08	0.07	0.03	0.03	0.00
95% confidence interval	0.4830 to 0.8270	0.5059 to 0.8441	0.7652 to 0.9948	0.3656 to 0.7344	0.4795 to 0.8505	0.5474 to 0.8726	0.6754 to 0.9596	0.8826 to 1.000	0.8994 to 1.000	1.000 to 1.000
P value	0.09	0.06	<0.0001	0.59	0.07	0.02	0.00	<0.0001	<0.0001	<0.0001
	CD8+PD-L1+ [%]									
	I vs. II	I vs. III	I vs. IV	II vs. III	II vs. IV	III vs. IV	I vs. HV	II vs. HV	III vs. HV	IV vs. HV
Area	0.52	0.99	1.00	0.98	1.00	0.67	1.00	1.00	1.00	1.00
Std. Error	0.09	0.01	0.00	0.02	0.00	0.09	0.00	0.00	0.00	0.00

95% confidence interval	0.3377 to 0.7073	0.9745 to 1.000	1.000 to 1.000	0.9367 to 1.000	1.000 to 1.000	0.4846 to 0.8504	1.000 to 1.000	1.000 to 1.000	1.000 to 1.000	1.000 to 1.000
P value	0.81	<0.0001	<0.0001	<0.0001	<0.0001	0.07	<0.0001	<0.0001	<0.0001	<0.0001
	CD19+PD-1+ [%]									
	I vs. II	I vs. III	I vs. IV	II vs. III	II vs. IV	III vs. IV	I vs. HV	II vs. HV	III vs. HV	IV vs. HV
Area	0.62	0.91	0.98	0.80	0.86	0.64	0.51	0.64	0.95	1.00
Std. Error	0.09	0.05	0.03	0.07	0.06	0.10	0.09	0.09	0.03	0.00
95% confidence interval	0.4466 to 0.7959	0.8173 to 0.9977	0.9249 to 1.000	0.6633 to 0.9367	0.7434 to 0.9816	0.4416 to 0.8284	0.3237 to 0.6913	0.4590 to 0.8160	0.8852 to 1.000	1.000 to 1.000
P value	0.19	<0.0001	<0.0001	0.00	<0.0001	0.14	0.94	0.14	<0.0001	<0.0001
	CD19+PD-L1+ [%]									
	I vs. II	I vs. III	I vs. IV	II vs. III	II vs. IV	III vs. IV	I vs. HV	II vs. HV	III vs. HV	IV vs. HV
Area	0.55	0.52	0.88	0.50	0.90	0.88	0.91	0.99	0.90	1.00
Std. Error	0.09	0.09	0.07	0.09	0.05	0.07	0.06	0.01	0.07	0.00
95% confidence interval	0.3616 to 0.7284	0.3339 to 0.7011	0.7484 to 1.000	0.3196 to 0.6854	0.7979 to 0.9971	0.7515 to 1.000	0.7996 to 1.000	0.9745 to 1.000	0.7685 to 1.000	1.000 to 1.000
P value	0.63	0.85	<0.0001	0.98	<0.0001	<0.0001	<0.0001	<0.0001	<0.0001	<0.0001
	sPD-1 serum concentration									
	I vs. II	I vs. III	I vs. IV	II vs. III	II vs. IV	III vs. IV	I vs. HV	II vs. HV	III vs. HV	IV vs. HV
Area	0.57	0.56	0.69	0.64	0.73	0.66	1.00	1.00	1.00	1.00
Std. Error	0.09	0.09	0.09	0.09	0.08	0.09	0.00	0.00	0.00	0.00
95% confidence interval	0.3897 to 0.7553	0.3767 to 0.7483	0.5158 to 0.8592	0.4574 to 0.8176	0.5683 to 0.8967	0.4862 to 0.8338	1.000 to 1.000	1.000 to 1.000	1.000 to 1.000	0.9893 to 1.000
P value	0.43	0.50	0.04	0.14	0.01	0.08	<0.0001	<0.0001	<0.0001	<0.0001
	sPD-L1 serum concentration									
	I vs. II	I vs. III	I vs. IV	II vs. III	II vs. IV	III vs. IV	I vs. HV	II vs. HV	III vs. HV	IV vs. HV
Area	0.58	0.56	0.69	0.64	0.73	0.66	1.00	1.00	1.00	1.00
Std. Error	0.09	0.09	0.09	0.09	0.08	0.09	0.00	0.00	0.00	0.00

95% confidence interval	0.3897 to 0.7553	0.3767 to 0.7483	0.5158 to 0.8592	0.4574 to 0.8176	0.5683 to 0.8967	0.4862 to 0.8338	1.000 to 1.000	1.000 to 1.000	1.000 to 1.000	0.9893 to 1.000
P value	0.43	0.50	0.04	0.14	0.01	0.08	<0.0001	<0.0001	<0.0001	<0.0001
	sPD-1 fluid concentration									
	I vs. II	I vs. III	I vs. IV	II vs. III	II vs. IV	III vs. IV				
Area	0.87	0.78	0.80	0.66	0.54	0.64				
Std. Error	0.06	0.07	0.07	0.09	0.10	0.09				
95% confidence interval	0.7633 to 0.9817	0.6344 to 0.9256	0.6531 to 0.9419	0.4825 to 0.8275	0.3491 to 0.7259	0.4580 to 0.8170				
P value	<0.0001	0.00	0.00	0.09	0.68	0.14				
	sPD-L1 fluid concentration									
	I vs. II	I vs. III	I vs. IV	II vs. III	II vs. IV	III vs. IV				
Area	0.78	0.78	0.73	0.54	0.55	0.56				
Std. Error	0.08	0.07	0.09	0.09	0.10	0.10				
95% confidence interval	0.6290 to 0.9260	0.6379 to 0.9271	0.5552 to 0.8998	0.3546 to 0.7204	0.3590 to 0.7385	0.3720 to 0.7480				
P value	0.00	0.00	0.01	0.68	0.60	0.52				

Abbreviations: HV- healthy volunteers

Supplementary Materials Table S2: Analysis of selected diagnostic test evaluation parameters

Parameters	CD4+PD-1+ [%]									
	I vs. II	I vs. III	I vs. IV	II vs. III	II vs. IV	III vs. IV	I vs. HV	II vs. HV	III vs. HV	IV vs. HV
PPV	0.500	0.400	0.300	0.350	0.286	0.450	0.750	0.750	0.750	0.750
NPV	0.500	0.400	0.300	0.350	0.263	0.450	0.750	0.750	0.750	0.750
Sensitivity	0.500	0.400	0.300	0.350	0.300	0.450	0.750	0.750	0.750	0.750
Specificity	0.500	0.400	0.300	0.350	0.250	0.450	0.750	0.750	0.750	0.750
Positive likelihood ratio (LR+)	1.000	0.667	0.429	0.538	0.400	0.818	3.000	3.000	3.000	3.000

Negative Likelihood Ratio (LR-)	1.000	1.500	2.333	1.857	2.800	1.222	0.333	0.333	0.333	0.333
Youden's J index	0.000	-0.200	-0.400	-0.300	-0.450	-0.100	0.500	0.500	0.500	0.500
Accuracy (ACC)	0.500	0.400	0.300	0.350	0.275	0.450	0.750	0.750	0.750	0.750
CD4+PD-L1+ [%]										
	I vs. II	I vs. III	I vs. IV	II vs. III	II vs. IV	III vs. IV	I vs. HV	II vs. HV	III vs. HV	IV vs. HV
PPV	0.500	0.381	0.286	0.400	0.286	0.350	0.737	0.750	0.750	0.750
NPV	0.474	0.400	0.263	0.400	0.263	0.350	0.750	0.750	0.750	0.750
Sensitivity	0.500	0.400	0.300	0.400	0.300	0.350	0.737	0.750	0.750	0.750
Specificity	0.474	0.381	0.250	0.400	0.250	0.350	0.750	0.750	0.750	0.750
Positive likelihood ratio (LR+)	0.950	0.646	0.400	0.667	0.400	0.538	2.947	3.000	3.000	3.000
Negative Likelihood Ratio (LR-)	1.056	1.575	2.800	1.500	2.800	1.857	0.351	0.333	0.333	0.333
Youden's J index	-0.026	-0.219	-0.450	-0.200	-0.450	-0.300	0.487	0.500	0.500	0.500
Accuracy (ACC)	0.487	0.390	0.275	0.400	0.275	0.350	0.744	0.750	0.750	0.750
CD8+PD-1+ [%]										
	I vs. II	I vs. III	I vs. IV	II vs. III	II vs. IV	III vs. IV	I vs. HV	II vs. HV	III vs. HV	IV vs. HV
PPV	0.429	0.429	0.300	0.524	0.429	0.400	0.650	0.714	0.714	0.750
NPV	0.421	0.400	0.300	0.526	0.421	0.400	0.650	0.737	0.737	0.750
Sensitivity	0.450	0.429	0.300	0.550	0.450	0.400	0.650	0.750	0.750	0.750
Specificity	0.400	0.400	0.300	0.500	0.400	0.400	0.650	0.700	0.700	0.750
Positive likelihood ratio (LR+)	0.750	0.714	0.429	1.100	0.750	0.667	1.857	2.500	2.500	3.000
Negative Likelihood Ratio (LR-)	1.375	1.429	2.333	0.900	1.375	1.500	0.538	0.357	0.357	0.333
Youden's J index	-0.150	-0.171	-0.400	0.050	-0.150	-0.200	0.300	0.450	0.450	0.500

Accuracy (ACC)	0.425	0.415	0.300	0.525	0.425	0.400	0.650	0.725	0.725	0.750
CD8+PD-L1+ [%]										
	I vs. II	I vs. III	I vs. IV	II vs. III	II vs. IV	III vs. IV	I vs. HV	II vs. HV	III vs. HV	IV vs. HV
PPV	0.500	0.250	0.250	0.263	0.211	0.429	0.571	0.550	0.750	0.750
NPV	0.500	0.250	0.250	0.250	0.238	0.421	0.579	0.550	0.750	0.750
Sensitivity	0.500	0.250	0.250	0.250	0.200	0.450	0.600	0.550	0.750	0.750
Specificity	0.500	0.250	0.250	0.263	0.250	0.400	0.550	0.550	0.750	0.750
Positive likelihood ratio (LR+)	1.000	0.333	0.333	0.339	0.267	0.750	1.333	1.222	3.000	3.000
Negative Likelihood Ratio (LR-)	1.000	3.000	3.000	2.850	3.200	1.375	0.727	0.818	0.333	0.333
Youden's J index	0.000	-0.500	-0.500	-0.487	-0.550	-0.150	0.150	0.100	0.500	0.500
Accuracy (ACC)	0.500	0.250	0.250	0.256	0.225	0.425	0.575	0.550	0.750	0.750
CD19+PD-1+ [%]										
	I vs. II	I vs. III	I vs. IV	II vs. III	II vs. IV	III vs. IV	I vs. HV	II vs. HV	III vs. HV	IV vs. HV
PPV	0.450	0.300	0.286	0.350	0.333	0.429	0.500	0.565	0.714	0.750
NPV	0.450	0.300	0.263	0.350	0.316	0.421	0.500	0.588	0.737	0.750
Sensitivity	0.450	0.300	0.300	0.350	0.350	0.450	0.500	0.650	0.750	0.750
Specificity	0.450	0.300	0.250	0.350	0.300	0.400	0.500	0.500	0.700	0.750
Positive likelihood ratio (LR+)	0.818	0.429	0.400	0.538	0.500	0.750	1.000	1.300	2.500	3.000
Negative Likelihood Ratio (LR-)	1.222	2.333	2.800	1.857	2.167	1.375	1.000	0.700	0.357	0.333
Youden's J index	-0.100	-0.400	-0.450	-0.300	-0.350	-0.150	0.000	0.150	0.450	0.500
Accuracy (ACC)	0.450	0.300	0.275	0.350	0.325	0.425	0.500	0.575	0.725	0.750
CD19+PD-L1+ [%]										
	I vs. II	I vs. III	I vs. IV	II vs. III	II vs. IV	III vs. IV	I vs. HV	II vs. HV	III vs. HV	IV vs. HV
PPV	0.476	0.500	0.300	0.500	0.300	0.300	0.700	0.739	0.700	0.750

NPV	0.474	0.500	0.300	0.500	0.300	0.300	0.700	0.824	0.700	0.750
Sensitivity	0.500	0.500	0.300	0.500	0.300	0.300	0.700	0.850	0.700	0.750
Specificity	0.450	0.500	0.300	0.500	0.300	0.300	0.700	0.700	0.700	0.750
Positive likelihood ratio (LR+)	0.909	1.000	0.429	1.000	0.429	0.429	2.333	2.833	2.333	3.000
Negative Likelihood Ratio (LR-)	1.111	1.000	2.333	1.000	2.333	2.333	0.429	0.214	0.429	0.333
Youden's J index	-0.050	0.000	-0.400	0.000	-0.400	-0.400	0.400	0.550	0.400	0.500
Accuracy (ACC)	0.475	0.500	0.300	0.500	0.300	0.300	0.700	0.775	0.700	0.750
sPD-1 serum										
	I vs. II	I vs. III	I vs. IV	II vs. III	II vs. IV	III vs. IV	I vs. HV	II vs. HV	III vs. HV	IV vs. HV
PPV	0.450	0.476	0.400	0.450	0.381	0.429	0.750	0.750	0.750	0.750
NPV	0.450	0.474	0.421	0.450	0.368	0.421	0.750	0.750	0.750	0.750
Sensitivity	0.450	0.500	0.421	0.450	0.400	0.450	0.750	0.750	0.750	0.750
Specificity	0.450	0.450	0.400	0.450	0.350	0.400	0.750	0.750	0.750	0.750
Positive likelihood ratio (LR+)	0.818	0.909	0.702	0.818	0.615	0.750	3.000	3.000	3.000	3.000
Negative Likelihood Ratio (LR-)	1.222	1.111	1.447	1.222	1.714	1.375	0.333	0.333	0.333	0.333
Youden's J index	-0.100	-0.050	-0.179	-0.100	-0.250	-0.150	0.500	0.500	0.500	0.500
Accuracy (ACC)	0.450	0.475	0.410	0.450	0.375	0.425	0.750	0.750	0.750	0.750
sPD-1 fluid concentration										
	I vs. II	I vs. III	I vs. IV	II vs. III	II vs. IV	III vs. IV	I vs. HV	II vs. HV	III vs. HV	IV vs. HV
PPV	0.333	0.350	0.350	0.600	0.476	0.429				
NPV	0.316	0.350	0.350	0.550	0.474	0.421				
Sensitivity	0.350	0.350	0.350	0.571	0.500	0.450				
Specificity	0.300	0.350	0.350	0.579	0.450	0.400				

Positive likelihood ratio (LR+)	0.500	0.538	0.538	1.357	0.909	0.750				
Negative Likelihood Ratio (LR-)	2.167	1.857	1.857	0.740	1.111	1.375				
Youden's J index	-0.350	-0.300	-0.300	0.150	-0.050	-0.150				
Accuracy (ACC)	0.325	0.350	0.350	0.575	0.475	0.425				
sPD-L1 serum										
	I vs. II	I vs. III	I vs. IV	II vs. III	II vs. IV	III vs. IV	I vs. HV	II vs. HV	III vs. HV	IV vs. HV
PPV	0.450	0.350	0.350	0.300	0.381	0.524	0.750	0.750	0.750	0.750
NPV	0.450	0.350	0.350	0.300	0.368	0.524	0.750	0.750	0.750	0.750
Sensitivity	0.450	0.350	0.350	0.300	0.400	0.524	0.750	0.750	0.750	0.750
Specificity	0.450	0.350	0.350	0.300	0.350	0.524	0.750	0.750	0.750	0.750
Positive likelihood ratio (LR+)	0.818	0.538	0.538	0.429	0.615	1.100	3.000	3.000	3.000	3.000
Negative Likelihood Ratio (LR-)	1.222	1.857	1.857	2.333	1.714	0.909	0.333	0.333	0.333	0.333
Youden's J index	-0.100	-0.300	-0.300	-0.400	-0.250	0.048	0.500	0.500	0.500	0.500
Accuracy (ACC)	0.450	0.350	0.350	0.300	0.375	0.524	0.750	0.750	0.750	0.750
sPD-L1 fluid concentration										
	I vs. II	I vs. III	I vs. IV	II vs. III	II vs. IV	III vs. IV	I vs. HV	II vs. HV	III vs. HV	IV vs. HV
PPV	0.350	0.350	0.381	0.476	0.476	0.476				
NPV	0.350	0.350	0.368	0.474	0.474	0.474				
Sensitivity	0.350	0.350	0.400	0.500	0.500	0.500				
Specificity	0.350	0.350	0.350	0.450	0.450	0.450				
Positive likelihood ratio (LR+)	0.538	0.538	0.615	0.909	0.909	0.909				

Negative Likelihood Ratio (LR-)	1.857	1.857	1.714	1.111	1.111	1.111	
Youden's J index	-0.300	-0.300	-0.250	-0.050	-0.050	-0.050	
Accuracy (ACC)	0.350	0.350	0.375	0.475	0.475	0.475	

Abbreviations: HV- healthy volunteers; PPV - positive predictive value; NPV - negative predictive value
